# Ecological stoichiometric characteristics of *Solanum rostratum* organs in different habitats

**DOI:** 10.3389/fpls.2025.1673588

**Published:** 2025-11-03

**Authors:** Lijun Hu, Juan Qiu, Xinping Zhu, Cai Ren, Kui Wang, Amanula Yimingniyazi

**Affiliations:** ^1^ College of Resources and Environmental, Xinjiang Agricultural University, Urumqi, China; ^2^ Xinjiang Key Laboratory of Ecological Adaptation and Evolution of Extreme Environment Organisms, College of Life Sciences, Xinjiang Agricultural University, Urumqi, China; ^3^ College of Bioscience and Resources Environment, Beijing University of Agriculture, Beijing, China

**Keywords:** alien invasive species, heterogeneous habitats, Solanum rostratum, organs, ecological stoichiometry

## Abstract

**Aims:**

Plant ecological stoichiometry focuses on the elemental content (such as carbon (C), nitrogen (N), and phosphorus (P)) in plant organs and its relationship with environmental factors and ecosystem functions. Alien invasive species ensure their rapid and efficient propagation by regulating their nutrient distribution, and they also influence soil physical and chemical properties by modifying the nutrient cycle and releasing allelochemicals, thus forming an environment conducive to their growth, reproduction, and diffusion. However, evidence on the ecological stoichiometry characteristics of the invasive plant *Solanum rostratum* and its invaded soils across different habitats in China, particularly the species’ nutrient utilization strategies in varying environments, is lacking.

**Methods:**

This study investigated *S. rostratum* in Xinjiang Province of China and analyzed the organ allocation of C, N, and P and stoichiometric characteristics across four distinct habitats (irrigation ditches, riparian zones, desert steppes, farmlands) through field surveys and controlled laboratory experiments. In addition, a conceptual framework elucidating *S. rostratum*’s invasion mechanisms, nutrient-use adaptations, and plant-soil feedback was established.

**Results:**

The results demonstrated that *S. rostratum* exhibits significant stoichiometric adaptation strategies in different habitats, thus providing a scientific foundation for understanding its invasion mechanisms and formulating effective control measures. The results indicated that significant differences in C, N, and P content occurred among the organs of *S. rostratum*, with a peak in reproductive tissues (flowers, fruits).

**Conclusion:**

This priority allocation to reproduction underscores an evolved strategy for optimizing fitness. Moreover, with changes in the invasion degree of *S. rostratum*, the soil nutrient cycle changed, with obvious differences in the responses of different habitats. This indicates that *S. rostratum* invasion not only affects its own growth but also affects the nutrient cycle of the whole ecosystem by changing the soil nutrient status. In addition, habitat type had a significant impact on the element accumulation of *S. rostratum*, and the quantitative relationship between plant and soil elements showed obvious habitat specificity. This phenomenon reflects the driving roles of environmental stress and resource availability on plant growth. *S. rostratum* adjusts its element absorption and distribution strategies under the environmental and resource conditions of different habitats as an adaptation to environmental changes. Therefore, *S. rostratum* adapts to heterogeneous environment or heterogeneous condition. Accordingly, differentiated control strategies tailored to its invasion characteristics in distinct habitats should be formulated to enhance its control efficiency.

## Introduction

The key factors for the large-scale expansion of alien invasive species to new areas are strong fecundity, extensive ecological adaptability, and soil nutrient regulation capacity (Palacio-LóPez and Gianoli, 2011). Soil is an important ecosystem component, and its physical and chemical properties in invasion areas impact the growth of invasive and native plants (Lin et al., 2021). Alien invasive species ensure their rapid and efficient propagation by regulating their nutrient distribution (Broadbent et al., 2018). In addition, they influence soil physical (Xiao et al., 2023; Ehrenfeld, 2003) and chemical properties by modifying the nutrient cycle and releasing allelochemicals, thus forming an environment that is conducive to their growth, reproduction, and diffusion but inhibits the growth and competitiveness of native plants (Redman et al., 2022). Plant ecological chemometrics is an important branch of ecological chemometrics that mainly focuses on the quantitative characteristics of elements distributed in plant organs as well as their relationship with environmental factors and ecosystem functions (Hu et al., 2022; Chen and Chen, 2021). Through field sampling and control experiments, researchers have explored the change law of plant chemical element stoichiometric characteristics, their response to global change, and their relationship with plant functional attributes, thereby promoting the rapid development of the field of plant ecological stoichiometry (Liu et al., 2024).

According to plant ecological stoichiometry, three core theoretical hypotheses have been proposed to explain the altered elemental ratios in invasive plant organs and invasion area soil: productivity-nutrient allocation hypothesis (Monson et al., 2022), N:P threshold hypothesis (Du et al., 2024), and stoichiometric homeostasis theory (Li et al., 2022). These hypotheses systematically illustrate the heterogeneity of resource allocation strategies of alien invasive species. The productivity-nutrient allocation hypothesis points out that plants will dynamically adjust the allocation of their own resources according to different environmental conditions to maximize resource utilization efficiency (Schönbeck et al., 2021). The N:P threshold hypothesis (Koerselman and Arthur, 1996) posits that nitrogen (N) and phosphorus (P) in plants typically maintain normal metabolism at a specific ratio. Thus, when the environmental N:P exceeds a critical threshold, P becomes the limiting factor, whereas when it falls below a certain threshold, N becomes the limiting factor. Different species exhibit varying adaptive capacities to N:P ratios, and such fluctuations may drive species succession. Tilman (1985) proposed that the essence of succession is the process of species competition and replacement driven by the temporal changes in the relative availability of limiting resources, rather than direct facilitation or inhibition among species, while Grime (1977) proposed that environmental changes will alter the composition of plant communities. Moreover, changes in the N:P ratio may lead to species replacement. For example, Dong et al. (2023) demonstrated that *Ammopiptanthus mongolicus* enhances its growth and development through efficient utilization of key nutrients such as N and P. Similarly, Zhao et al. (2025) revealed that *Xanthium italicum* dynamically adjusts its resource allocation patterns in response to environmental heterogeneity.Stoichiometric homeostasis theory is a key framework for understanding biological environmental interactions, and it states that organisms (plants and animals) will maintain a relatively constant element ratio in environments with different resources via physiological, behavioral, and evolutionary factors (Sardans et al., 2021).

*Solanum rostratum* is an annual herb belonging to the Solanaceae family, and it is native to north Mexico and the southwestern United States. In China it was first recorded in Hong Kong in 1895 (Wang et al., 2020). However, it is also distributed in various habitats in Xinjiang, China (Jianxiao et al., 2013), and its growth status exhibits habitat heterogeneity (Qiu et al., 2013). The plant is covered with strong spines and presents high toxicity based on the contents of solanine and other toxic substances, which induce respiratory paralysis or death in humans and livestock. *S. rostratum* also has strong reproductive ability, and the fruit bearing capacity of general plants can reach 20000 (Yu and Tan, 2007). Moreover, the fruit easily attaches to animal fur, human clothing, or farm tools for transmission. Therefore, it has been listed as a Category 1 malignant invasive plant species on the List of Key-Managed Invasive Alien Speciesin China.

Research on *S. rostratum* in China and worldwide mainly focuses on its diffusion mechanisms (Sayit et al., 2019), distribution areas (Zhong et al., 2009), allelopathy (Sayit et al., 2019), and reproductive characteristics (Yu and Tan, 2007). However, evidence on the ecological stoichiometry characteristics of *S. rostratum* and its invaded soils across different habitats, particularly the species’ nutrient utilization strategies in varying environments, is still lacking in the published literature. Therefore, this study focused on the invasive plant *S. rostratum* as the main research material and selected four typical invasion sites around Urumqi and Changji in Xinjiang as the research areas. By studying the ecostoichiometric characteristics of each organ (root, stem, leaf, flower, and fruit), the soil characteristics under four different habitats (irrigation ditch, riparian zone, desert steppe, and farmland), and the different invasion degrees (uninvaded control plots, low-invasion-intensity plots, moderate-invasion-intensity plots, and high-invasion-intensity plots), this study established a conceptual framework to elucidate *S. rostratum*’s invasion mechanisms, nutrient-use adaptations, and plant-soil feedback.

Field investigations prior to the experiment revealed that *S. rostratum* was not observed around Urumqi in early May but began to gradually emerge by mid-June. In early July, although the plants continued to grow, their distribution range remained limited and insufficient to meet the sampling requirements of this study. By early August, *S. rostratum* had extensively invaded areas in Urumqi and Changji, colonizing various habitats with frequent human activities, such as irrigation ditches, riparian zones, desert steppes, farmlands, and oases. Significant differences were observed in the distribution patterns and growth status (e.g., plant height, leaf area, and other morphological indicators) of *S. rostratum* across different habitats. In irrigation ditches and riparian zones, the species predominantly exhibited a punctate distribution, whereas in farmlands and desert steppes, it mainly occurred in sheet-like formations and was concentrated in areas with frequent human and animal activities (such as roadsides and ridges). The primary morphological variations among habitats were reflected in plant height and leaf morphology. Significant differences were observed in the distribution pattern and growth status (such as the plant height, leaf area, and other morphological indicators) of *S. rostratum* in different habitats. Based on these field observations and previous research findings, we propose the following research hypotheses:Theory of Ecological Stoichiometric Homeostasis, Reproduction-First Strategy, Organ-Specific Allocation Hypothesis, Habitat-Specific Response Hypothesis, Plant-Soil Feedback Hypothesis. The primary objectives are to (1) explore the change trends in the ecostoichiometric characteristics of the different organs of *S. rostratum* in different habitats; (2) determine how the resource allocation strategy among the organs drives the successful invasion of *S. rostratum*; and (3) identify the habitat specificity of the mutual feeding mechanism between soil nutrient cycling and *S. rostratum* invasion.

## Materials and methods

### Study area

Research plots were established in the Midong and Shuimogou Districts of Urumqi and within the large-scale expansion area of *S. rostratum* around Sangong town of Changji City (**Table 1**). Urumqi has a temperate continental arid climate, undulating terrain, and vast mountainous areas. The study area receives ≈200 mm annual precipitation but experiences potential evaporation of 2000–2500 mm/yr, indicating extreme aridity. The habitat types are diverse. In addition to the widely distributed desert steppe vegetation, grassland and alpine vegetation are also present. The field investigation indicated that *S. rostratum* is widely distributed across multiple counties and diverse habitats in Urumqi. Changji City features a typical continental arid climate within the middle-temperate zone. Topographic influences drive pronounced north–south climatic variations, with the southern areas receiving higher summer rainfall and the northern areas exhibiting distinct desert climate characteristics. Changji Prefecture benefits from abundant sunshine (annual duration: 2,700 h) and favorable thermal conditions (≥10°C annual accumulated temperature: 3,450°C days, mean annual temperature: 6.8°C, and January temperature: −15.6°C) The region experiences low precipitation (190 mm/year) but high potential evaporation (1,800–2,200 mm/year), resulting in a severe moisture deficit.

**Table 1:** Sampling plot information.

Habitat type	Altitude	Place	Position	Main companion species
Irrigation ditch	899m	Shuimogou District, Urumqi	43.820805°N, 87.694827°E	*Artemisia argyi* *Atriplex centralasiatica* *Echinochloa colona* *Eragrostis pilosa* *Sedum ewersii* *Lactuca serriola*
Riparian zone	845m	Midong District, Urumqi	43.925376°N, 87.802188°E	*Polygonum aviculare* *Atriplex patens* *Salsola collina* *Seriphidium terrae-albae* *Artemisia argyi* *Peganum harmala* *Ceratocarpus arenarius* *Descurainia sophia* *Lactuca serriola* *Atriplex laevis*
Desert steppe	1008m	Shuimogou District, Urumqi	43.821201°N, 87.778304°E	*Peganum harmala* *Onopordum acanthium* *Puccinellia distans* *Stipa capillata* *Artemisia argyi* *Tamarix ramosissima* *Salsola affinis* *Atriplex patens* *Euphorbia humifusa* *Heliotropium europaeum*
Farmland	726m	Sangong Town, Changji	43.897518°N, 87.202757°E	*Artemisia argyi* *Polygonum aviculare* *Atriplex patens* *Onopordum acanthium* *Setaria viridis* *Ceratocarpus arenarius* *Peganum harmala* *Convolvulus arvensis* *Sophora alopecuroides* *Heliotropium europaeum*

### Sampling design

Based on a combination of the grading method used by Xiao et al. (2023) and distribution range of *S. rostratum* in Xinjiang, four habitat types (irrigation ditch, riparian zone, desert steppe, and farmland) were sampled to investigate *S. rostratum*. Experimental plots were set up for each habitat according to the different invasion pressures. Considering the plant height, crown width, and other morphological indices of *S. rostratum*, the area of each quadrat was 2 m×2 m. According to the different coverages of *S. rostratum*, the plots were categorized as the control (0% coverage), low invasion (<35% coverage), moderate invasion (35%–75% coverage), and high invasion plots (>75% coverage). Each plot was divided into 12 test quadrats, with 3 control quadrats, 3 low-invasion quadrats, 3 moderate-invasion quadrats, and 3 high-invasion quadrats.

To exclude temporal confounding effects, plant and soil samples of *S. rostratum* were simultaneously collected in early August 2024. During sampling, *S. rostratum* was in bloom in three sampling sites (irrigation ditch, riparian zone, and desert steppe). The flowers were brightly colored and showed a relatively consistent morphology, and the petals were spread out and fully open. The leaves exhibited a healthy green color, and their size and shape were normal, indicating a good growth trend. At the farmland sampling site, *S. rostratum* was at the fruiting stage and showed a stable overall growth trend and extremely distinct fruit characteristics. The fruits appeared light yellow, and the number of leaves was lower than that in the other three habitats. During collection, all herbaceous plant species in each quadrat were investigated, basic information on the sampling site and main companion species, were recorded (**Table 1**), and the coverage, plant height, and chlorophyll content of *S. rostratum* were measured (**Table 2**). Plant samples included five whole healthy plants of *S. rostratum* collected from each sample plot. After collection, plants from the different quadrats were placed into sample bags and marked. The five-point soil sampling method was used, in which the sampling points were arranged in the shape of a plum blossom. In addition, soil from each quadrat was collected separately, placed into a sample bag, and marked.

**Table 2:** Basic information of *S. rostratum* communities in different habitat types.

Habitat type	Degree of invasion	Coverage (%)	Plant height (cm)	Chlorophyll (mg/g)
Irrigation ditch	L	15 ± 4.36	31.57 ± 14.08^Aa^	52.07 ± 9.48^Aa^
	M	44.33 ± 17.93	47.89 ± 26.18^ABb^	46.36 ± 7.55^Ab^
	H	81 ± 3.61	47.74 ± 18.62^ABb^	47.61 ± 5.93^Ab^
Riparian zone	L	22.67 ± 4.04	25.4 ± 11.53^Aa^	48.55 ± 6.85^Aa^
	M	61.67 ± 20.21	38.13 ± 16.79^ABab^	52.27 ± 7.47^Bb^
	H	75.33 ± 4.51	27.2 ± 10.97^Cb^	53.48 ± 6.38^Bab^
Desert steppe	L	18.33 ± 7.64	24.4 ± 13.74^Aa^	50.55 ± 10.94^Aa^
	M	48.33 ± 10.41	31.46 ± 8.11^Aa^	55.30 ± 8.16^Ba^
	H	88.33 ± 2.89	40.58 ± 7.82^Ab^	51.63 ± 8.91^ABb^
Farmland	L	16.67 ± 2.89	14.39 ± 7.54^Ba^	59.79 ± 12.54^Ba^
	M	45 ± 10	44.17 ± 19.61^Bb^	52.95 ± 10.44^Bb^
	H	75.67 ± 1.15	50.73 ± 13.02^Bb^	55.49 ± 9.77^Bb^

capital letters represent significant differences in the same degree of invasion in different habitats (P<0.05), and small letters represent significant differences in different degrees of invasion in the same habitat (P<0.05).

The element contents of the samples were determined in the laboratory. For the plant sample analysis, specimens from each quadrat were dissected into organs (roots, stems, leaves, flowers, and fruits) and labeled, and fresh tissues were enzyme-inactivated (105 °C, 15 min) to halt metabolic activity and then dehydrated at 55 °C to a constant weight. Dry plant samples were ground and passed through a 100-mesh sieve (<0.149 mm), organ-specific analyses were performed, including organic carbon (potassium dichromate titration), total nitrogen (H_2_O_2_ digestion-Kjeldahl method), total phosphorus (H_2_O_2_ digestion-molybdenum antimony anti-spectrophotometry), and total chlorophyll (Top Cloud-Agri Chlorophyll Meter TYS-B). Multiple specific wavelengths were selected, and the absorption degrees at these wavelengths were measured and converted into the total content of chlorophyll a and chlorophyll b. For the soil sample analysis, crushed soils were divided into two subsamples: 1) air-dried soils, which were sieved (<2 mm) and tested for pH (NY/T 1121.2–2006 standard), moisture content (oven-drying method), ammonium nitrogen, and nitrate nitrogen (KCl extraction-flow analyzer); and 2) field-moist soils, which were sieved (<2 mm) and tested for total carbon (potassium dichromate oxidation), total nitrogen (H_2_O_2_ digestion-Kjeldahl method), and total phosphorus (molybdenum antimony anti-spectrophotometry).

### Statistical analyses

Excel 2024 was used to sort the data, and SPSS 27.0 was used to perform statistical analyses of each index. The mean and standard deviation (mean ± SD) were calculated. One-way ANOVA assessed statistically significant differences in plant height among groups and chlorophyll under different habitats and different invasion degrees based on the least significant difference (LSD) analysis method, and the significance level of the stoichiometric characteristics between organs and habitats was set at P < 0.05. The weighted average of the biomass was used to calculate the weight and stoichiometric ratios of the element content in each organ of *S. rostratum*, and whole-plant elemental concentrations (C, N, and P) and stoichiometric ratios were derived as biomass-weighted averages. The correlations among plant indices were discussed through correlation analysis, and the main effects and interactions of organ type, invasion degree, and habitat on *S. rostratum* stoichiometry (C, N, P, C:N, C:P, and N:P) were assessed via generalized linear models (GLMs).

Soil properties (C, N, P, C:P, C:N, N:P, pH, water content, ammonium nitrogen, and nitrate nitrogen contents) were analyzed for significant differences based on the habitat and invasion degree using two-way ANOVA (α = 0.05). Pairwise relationships among soil variables were assessed via Pearson correlation analysis. GLMs with a Gaussian distribution were used to quantify the main effects and invasion × habitat interactions on soil stoichiometry. Finally, principal component analysis was carried out on the content and measurement ratio of elements between plants and soil in each habitat. The load on the principal component axis of each index was calculated, and the correlation between the first two principal components of plants and soil was further analyzed. Data processing was performed in SPSS Statistics 23.0 (IBM Corp.). Graphical visualizations and statistical analyses were conducted using OriginPro 2022 (OriginLab Corporation).”

## Results

The relationship between plant height and invasion degree varied in the different habitats. In the irrigation ditch habitat, plant height showed relatively little change during low-degree, medium-degree, and high-degree invasions. However, in the desert steppe habitat, plant height increased to a certain extent as the invasion degree increased. In the riparian zone habitat, plant height initially increased and then decreased as the invasion degree increased. Under the same invasion degree, significant differences in plant height were observed across the different habitats. During low-degree invasion, significantly higher plant heights were observed in the irrigation ditch, riparian zone, and desert steppe than in the farmland. During high-degree invasion, significantly lower plant heights were observed in the irrigation ditch than in the other three habitats. The chlorophyll content of *S. rostratum* leaves also showed different levels under the different habitats and invasion degrees. As the invasion degree increased, the chlorophyll content of *S. rostratum* in the irrigation ditch, desert steppe, and farmland showed a significant and gradual decreasing trend, while that in the riparian zone showed a significant increasing trend. Under the different invasion degrees, the chlorophyll value was the lowest in the irrigation dtch and highest in the farmland (**Table 1**).

**Figure 1:**
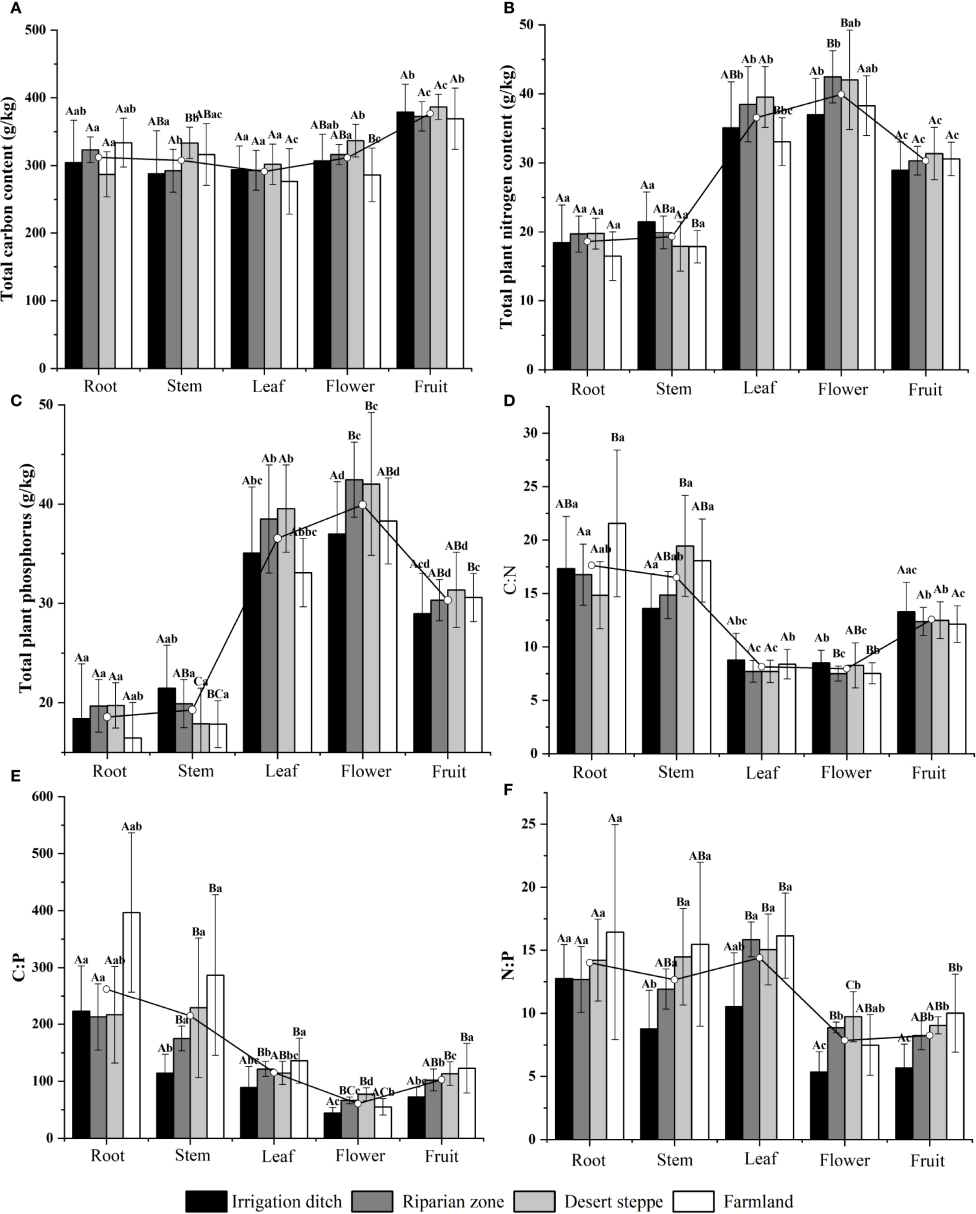
Contents and stoichiometric ratios of C, N, and P in different organs of *S. rostratum* across various habitats. Uppercase letters indicate significant differences between the same organ in different habitats (P < 0.05), lowercase letters indicate significant differences among different organs in the same habitat (P < 0.05).The unit of measurement for **(A–C)** is g/kg. **(A)** compares the carbon content across different organs (root, stem, leaf, flower, and fruit) of *S. rostratum*; **(B)** compares the nitrogen content among organs; **(C)** compares the phosphorus content among organs; **(D)** shows the C:N ratio across organs; **(E)** shows the C:P ratio across organs; and **(F)** shows the N:P ratio across organs.

Univariate statistics of elemental concentrations (C, N, and P) and these core elements stoichiometric ratios (C:N, C:P, and N:P) in various organs of *S. rostratum* are shown in **Figure 1**. In the organs of *S. rostratum*, the content of C was ordered fruit > flower > root > stem > leaf; the content of N was ordered flower > leaf > fruit > stem > root, and the content of P was ordered flower > fruit > leaf > stem > root. Although the C content was highest in the fruit, high contents were observed in the flowers and stems in the desert steppe. N and P accumulation in the flowers peaked in the riparian and desert-steppe habitats, whereas N and P accumulation peaked in the leaves and flowers in the irrigation-ditch ecosystems. Specifically, the carbon content in the fruit was 29.57% higher than that in the leaves, while the carbon content in the flowers was 7.95% higher than that in the leaves. The nitrogen content in the flowers was 119.18% higher than that in the roots, and the nitrogen content in the fruit was 62.54% higher than that in the roots. The phosphorus content in the flowers was 265.52% higher than that in the roots, and the phosphorus content in the fruit was 165.73% higher than that in the roots. Organ-specific stoichiometry revealed that the C:N and C:P ratios strictly decreased from the roots (maxima) to flowers (minima) in the sequence root > stem > fruit > leaf > flower. The N:P exhibited a different trend and decreased from the leaves (maxima) to flowers (minima) in the sequence leaf > root > stem > fruit > flower. This highlights that flowers represent elemental investment hotspots despite ratio minimization. Among the habitats, the leaf C:N ratios exhibited a decreasing gradient of irrigation ditches > farmland > riparian zones > desert steppe, whereas the leaf C:P ratios declined along a gradient of farmland > riparian zones > desert steppe > irrigation ditches.

**Figure 2:**
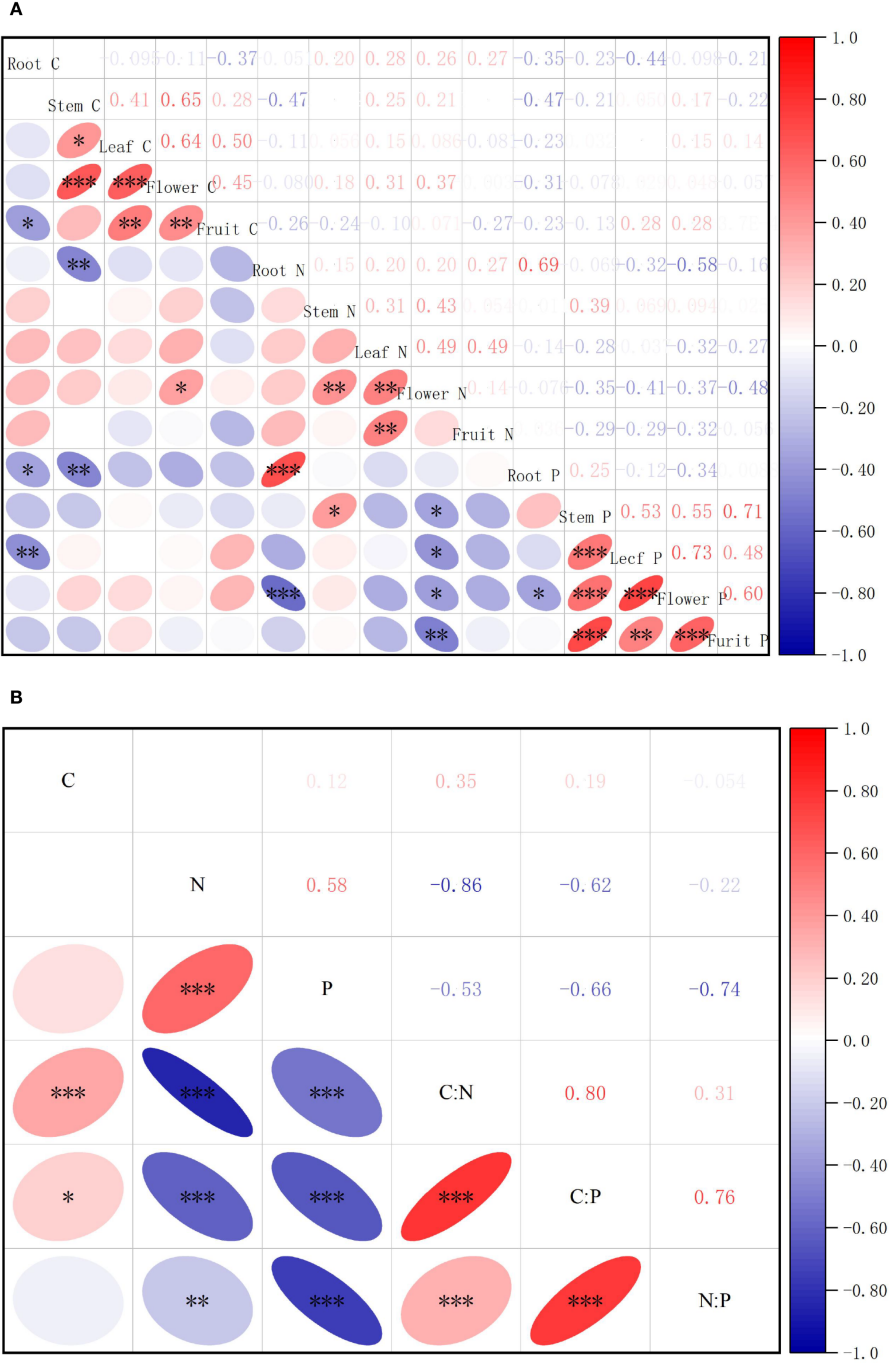
Correlation analysis of element contents and stoichiometric characteristics in various organs of *S. rostratum.* **(A)** shows the correlation analysis of C, N, P, and their stoichiometric ratios in various organs of S. rostratum, while **(B)** presents the correlation analysis of C, N, P, and their stoichiometric ratios in the whole plant of *S. rostratum.* * P<0.05, **P<0.01, ***P<0.001.

Correlation analysis of the elemental concentrations across *S. rostratum* organs (**Figure 2**) demonstrated significant stoichiometric linkages. Among 105 elemental pairs, 28 pairs (26.67%) exhibited statistically significant correlations, including 14 positive and 9 negative inter-organ relationships versus 3 positive and 2 negative intra-organ correlations. This pattern indicates stronger stoichiometric coordination between organs than within organs under habitat heterogeneity. Correlation analysis of *S. rostratum* revealed highly synergistic accumulation of N and P across organs (P < 0.01), whereas C did not show significant associations with other elements. C showed a highly significant positive correlation with the C:N ratio (P < 0.001) and a significant positive correlation with the C:P ratio (P < 0.05). N exhibited robustly significant negative correlations with both the C:N and C:P ratios (P < 0.001) and a significant negative correlation with the N:P ratio (P < 0.01). P demonstrated robustly significant negative correlations with all three stoichiometric ratios: C:N, C:P, and N:P (P < 0.001). Furthermore, strong positive intercorrelations were observed among all ratios: C:N, C:P, and N:P (P < 0.001), indicating high internal coordination within the stoichiometric framework.

The contents of C, N, and P and their stoichiometric ratios (C:N, C:P, and N:P) in *S. rostratum* were differentially influenced by the organ type, invasion degree, habitat, and their single, double, and triple interactions (**Table 3**). Specifically, organ type exerted a robustly significant effect (P < 0.01) on the C, N, and P content, as well as on the C:N, C:P, and N:P ratios. Invasion degree significantly affected the C and N content (P < 0.05) and exerted a robustly significant effect on the C:P and N:P ratios (P < 0.01). Habitat exerted a robustly significant effect on the N and P content and C:P and N:P ratios (P < 0.01) and a significant effect on the C:N ratio (P < 0.05). Organ type and invasion degree interactively drove robustly significant variations (P < 0.01) in P content and stoichiometric ratios and significant variations (P < 0.05) in C and N content. Organ type and habitat interaction robustly and significantly affected the P content and stoichiometric ratios (P < 0.01), while exerting significant effects on C and N content (P < 0.05). Robustly significant effects (P < 0.01) of the triple interaction (organ × invasion × habitat) were observed on the C:P ratio.

**Table 3:** GLM analysis of habitat and organ elfects on C, N, P contents and their stoichiometric characteristics (F value).

Independent variable	Organ	Degree of invasion	Habitat	Organ * invasion degree	Organ * Habitat	Interaction
C content	23.99^**^	3.55^*^	1.36	0.89	2.05^*^	0.74
N content	237.00^**^	3.89^*^	7.34^**^	1.62	2.30^*^	1.07
P content	128.60^**^	0.22	31.06^**^	3.25^**^	3.91^**^	1.41
C:N	98.69^**^	2.18	3.04^*^	2.77^**^	4.08^**^	0.76
C:P	64.43^**^	10.15^**^	16.83^**^	6.77^**^	5.09^**^	2.91^**^
N:P	37.36^**^	6.37^**^	18.72^**^	3.07^**^	1.63	1.36

* P<0.05, **P<0.01

**Figure 3:**
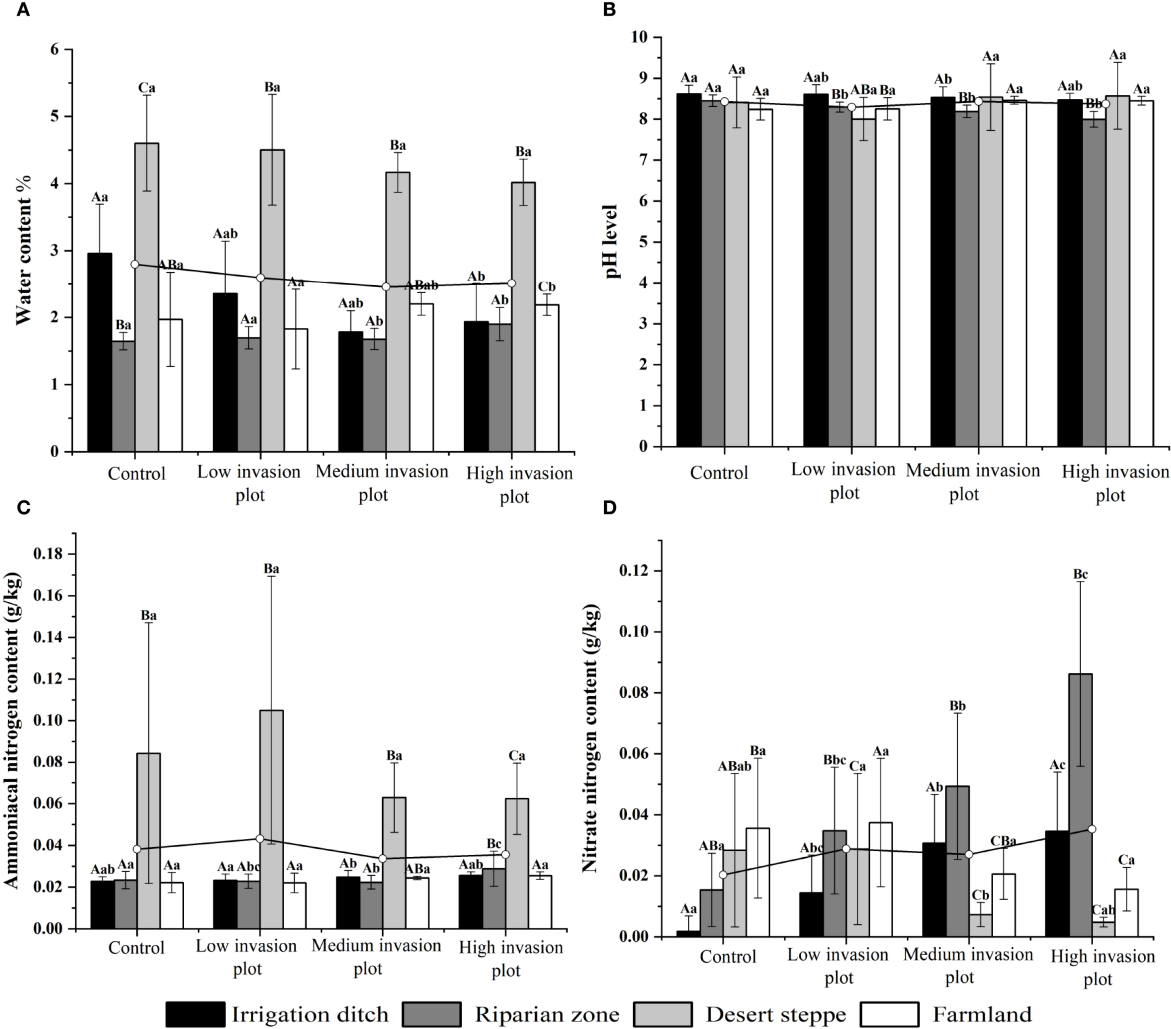
Soil moisture, pH, ammonium nitrogen, and nitrate nitrogen contents across different habitatszz. Uppercase letters indicate significant differences between the same organ in different habitats (P < 0.05), lowercase letters indicate significant differences among different organs in the same habitat (P < 0.05). **(A)** shows the variations in soil water content across different habitats under varying invasion degrees, with measurement units in %. **(B)** illustrates the changes in soil pH across different habitats under varying invasion degrees. **(C)** displays the variations in soil ammonium nitrogen content across different habitats under varying invasion degrees, with measurement units in g/kg. **(D)** presents the changes in soil nitrate nitrogen content across different habitats under varying invasion degrees, with measurement units in g/kg.

**Figure 4:**
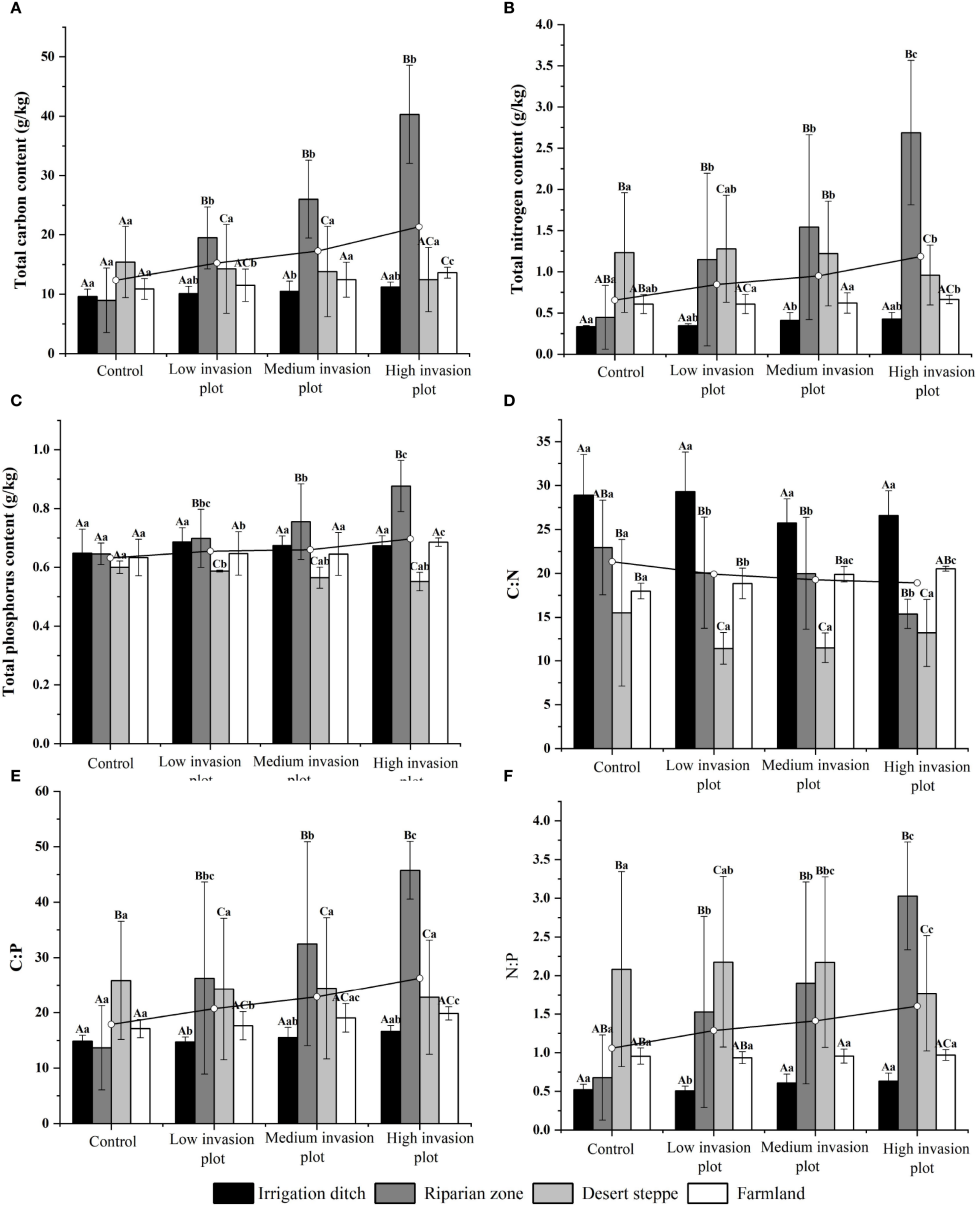
Soil C, N, P contents and their stoichiometric ratios in different habitats. Uppercase letters indicate significant differences between the same organ in different habitats (P < 0.05), lowercase letters indicate significant differences among different organs in the same habitat (P < 0.05). **(A)** shows the variation in soil carbon content across different habitats under varying invasion degrees. **(B)** illustrates the variation in soil nitrogen content across different habitats under varying invasion degrees. **(C)** presents the variation in soil phosphorus content across different habitats under varying invasion degrees, with measurement units in g/kg. **(D)** displays the variation in the soil C:N ratio across different habitats under varying invasion degrees. **(E)** depicts the variation in the soil C:P ratio across different habitats under varying invasion degrees. **(F)** demonstrates the variation in the soil N:P ratio across different habitats under varying invasion degrees.

A descriptive analysis was conducted on soil physicochemical properties under different invasion degrees of *S. rostratum* across various habitats. These properties included pH, moisture content, ammonium nitrogen, nitrate nitrogen, the contents of C, N, and P, and their stoichiometric ratios. Across the habitats, the soil moisture content under different invasion levels generally exhibited an initially decreasing and then increasing trend (**Figure 3**). The pH value showed some variations but a relatively small magnitude of change. The ammonium nitrogen content displayed an initially increasing and then decreasing trend, with the most significant change observed in the desert steppe, where it decreased by 20.32%. The nitrate nitrogen content in irrigation ditches and riparian zones increased significantly with increasing invasion intensity, rising by 244.56% and 700.08%, respectively. In contrast, the nitrate nitrogen content in the desert steppe showed a significant decreasing trend (P < 0.05) with increasing invasion degree, declining by 40.37%. Across the habitats, the contents of soil C, N, and P and soil C:P and N:P ratios generally increased with increasing invasion intensity (**Figure 4**). From the control to heavily invaded plots, the parameters increased by 73%, 104.32%, 10.30%, 37.03%, and 59.15%. In contrast, the soil C:N ratio decreased with increasing invasion degree, declining by 19.73%.

**Figure 5:**
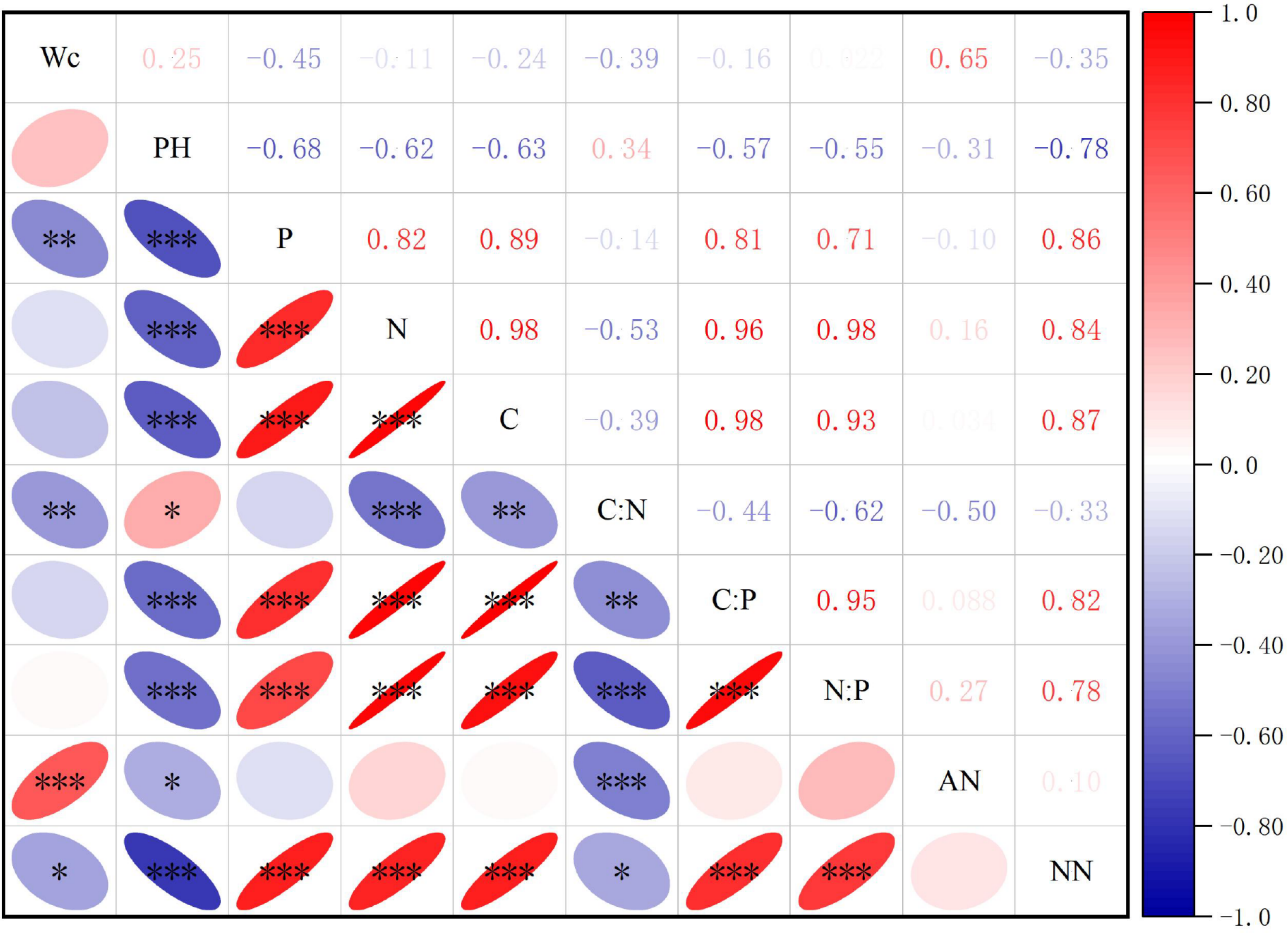
Correlation analysis of soil element contents and ttheir stoichiometric characteristics across different habitats. Wc represents soil water content; pH is an indicator of soil acidity or alkalinity; C, N, and P denote soil carbon, nitrogen, and phosphorus contents, respectively; C:N, C:P, and N:P represent the ratios of carbon to nitrogen, carbon to phosphorus, and nitrogen to phosphorus, respectively; AN stands for soil ammonium nitrogen content, NN refers to soil nitrate nitrogen content. * P<0.05, **P<0.01, ***P<0.001.

The correlation analysis of the contents of various soil elements (**Figure 5**) showed that of the 45 pairs of soil chemical elements, water content, pH value, ammonium-nitrogen content, and nitrate-nitrogen content interactions, 33 pairs reached a significant level, accounting for 73.33%. Correlation analysis of soil indicators across the different invasion degrees of *S. rostratum* revealed that the soil moisture content showed significantly negative correlations with the soil P and C:N (P < 0.01), negative correlations with nitrate nitrogen (P < 0.05), and a robustly significant positive correlation with ammonium nitrogen (P < 0.001). Soil pH demonstrated robustly significant negative correlations with soil P, N, C, ammonium nitrogen, C:P, and N:P (P < 0.001), a positive correlation with C:N, and a positive correlation with ammonium nitrogen (P < 0.05). Regarding elemental interactions, P, N, and C exhibited robustly significant positive pairwise correlations (P < 0.001) and robustly significant positive correlations with C:P, N:P and nitrate nitrogen (P < 0.001). In addition, N and C were robustly significantly negatively correlated with C:N. For the stoichiometric relationships, C:N displayed robustly significant negative correlations with C:P, N:P, and ammonium nitrogen (P < 0.001) and a negative correlation with nitrate nitrogen (P < 0.05); C:P exhibited robustly significant positive correlations with N:P and nitrate nitrogen (P < 0.001); and N:P was robustly significant positive correlations with nitrate nitrogen.

Under varying invasion degrees of *S. rostratum*, the soil elements (C, N, and P) and their stoichiometric ratios were differentially modulated by invasion degree, habitat, and their interactions (**Table 4**). Regarding single-factor effects, both invasion degree and habitat exerted robustly significant effects on C and N (P < 0.01); invasion degree significantly affected P (P < 0.05), while habitat showed a robustly significant impact on P (P < 0.01), with habitat demonstrating a stronger overall influence on C, N, and P and C:P and N:P. For stoichiometric ratios, invasion degree significantly influenced C:N and N:P (P < 0.05) and had a robustly significant effect on C:P (P < 0.01), whereas habitat significantly affected the C:N ratio (P < 0.05). For interactions, the invasion degree and habitat interaction generated robustly significant effects on C, N, P, C:P, and N:P (P < 0.01), and exerted robustly significant interactive effects on all three stoichiometric ratios (C:N, C:P, and N:P; P < 0.01).

**Table 4:** GLM analysis of habitat and organ elfects on C, N, P,K contents and their stoichiometric characteristics (F value).

Independent variable	Degree of invasion	Habitat	Interaction	Independent variable	Degree of invasion	Habitat	Interaction
C content	7.93^**^	73.10^**^	12.02^**^	C:N	3.597^*^	26.73^**^	1.38
N content	5.52^**^	51.545^**^	9.51^**^	C:P	5.146^**^	43.36^**^	9.43^**^
P content	4.22^*^	63.30^**^	7.83^**^	N:P	3.359^*^	34.92^**^	7.92^**^

* P<0.05, **P<0.01

**Figure 6:**
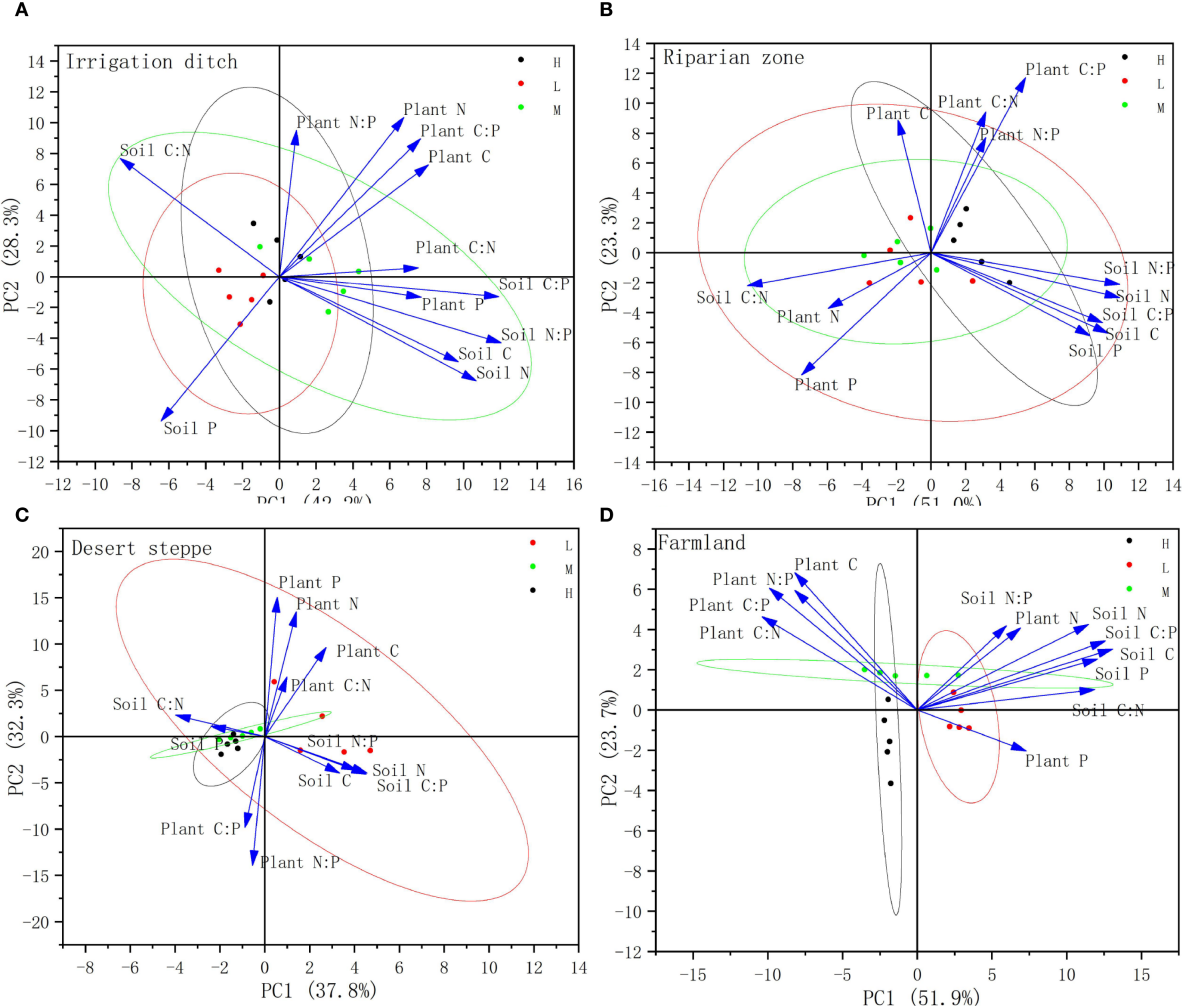
PCA analysis of C, N, P contents and their stoichiometric ratios in *S. rostratum*-soil systems under different invasion levels. **(A)** denotes the PCA analysis diagram for the Irrigation Ditch; **(B)** denotes the PCA analysis diagram for the Riparian Zone; **(C)** denotes the PCA analysis diagram for the Desert Steppe; **(D)** denotes the PCA analysis diagram for the Farmland.

Principal component analysis (PCA) was conducted separately on *S. rostratum* and the soil C, N, and P concentrations and stoichiometric ratios across four habitat types (**Figure 6**). The results are presented in **Figure 6**. Two principal components were extracted for all habitats: irrigation ditches explained 42.2% of the variance for PC1 and 28.3% for PC2 (cumulative 70.5%); riparian zones explained 51.0% of the variance for PC1 and 23.3% for PC2 (cumulative 74.3%); desert steppe explained 37.8% of the variance for PC1 and 32.3% for PC2 (cumulative 70.1%); while farmland displayed explained 51.9% for the variance for PC1 and 23.7% for PC2 (cumulative 75.6%). All cumulative variance values exceeded the 60% reliability threshold.

In the irrigation ditches habitat, the soil C, N, C:P, and N:P and plant P and C:N were positively correlated with each other. Conversely, the soil C:N was negatively correlated with the plant P and C:N and soil C, N, C:P, and N:P. In the riparian habitat, the soil C:N and plant N and P contents were negatively correlated with the plant C:N, C:P, and N:P and soil C, N, P, C:N, and N:P. In the desert steppe, the soil P content and C:N were negatively correlated with the plant P content and C:N. In the farmland habitats, the plant C:N was negatively correlated with the plant P and soil N, P, and C:N.

## Discussion

The C, N, and P contents and distribution ratio of elements in the different organs of *S. rostratum* showed certain differences. Overall, the C content in the fruit of *S. rostratum* was significantly higher than that in the other organs. Less C was accumulated in the leaves, stems, roots, and flowers, more C was accumulated in the fruit, and more P was accumulated in the flowers and fruits, which is related to the plant’s growth strategy in a low-resource environment (Zhang et al., 2019). This result supports the productivity–nutrient allocation hypothesis. After balancing reproductive and vegetative growth, invasive species allocate a portion of resources and nutrients to the fruit to meet the developmental needs of reproductive organs. This finding is consistent with the conclusion of Sun et al. (2023), who showed that the components of *S. rostratum* have different allometric growth laws and the proportion of nutrients allocated by plants to reproductive components increases as the biomass increases.

Robustly different C, N, and P elemental contents and C:P, C:N, and N:P stoichiometric ratios were observed across the organs of *S. rostratum* among the different habitats, as characterized in **Figure 1**. Specifically, the leaf C:N and C:P ratios, which are key indicators reflecting plant carbon assimilation capacity and nutrient use efficiency, demonstrated habitat-dependent variations. Although the leaf C:N ratios showed no significant differences among habitats, the leaf C:P ratios varied significantly (P < 0.05). This indicates that plant nutrient utilization strategies are regulated by habitat conditions, with higher C:P values directly signifying greater nutrient use efficiency. This result may be related to the resource pressure of different environments, which drives the adaptation strategies of the organs of *S. rostratum*. Implications for Its Competitive Success in Colonization. The growth adaptation strategies of *S. rostratum* form a synergistic system that provides multiple advantages for colonization and competition across diverse habitats. Its morphological plasticity enables effective light competition through height adjustment, while physiological plasticity optimizes resource-use efficiency by regulating chlorophyll content and elemental stoichiometry. Crucially, a reproduction-first strategy directs premium resources (N and P) to reproductive organs, ensuring high reproductive output and invasion potential. Coupled with its generalist adaptation to various nutrient limitations (N, P, or co-limitation), this species thrives in heterogeneous environments. Henn et al. (2018) showed that plant growth adaptability changes with changes in habitat. This is consistent with our findings, which demonstrated that *S. rostratum* possesses a remarkable capacity for resource allocation. Therefore, the theory of ecological stoichiometric homeostasis, wherein plants maintain relatively stable internal elemental ratios through physiological regulation and evolutionary adaptation, was validated.

The leaf N:P ratio serves as a critical parameter for diagnosing plant nutrient limitation status (Li et al., 2018), thus playing a decisive role in determining community structure and ecosystem functioning. Data across the four habitats revealed that the leaf N:P was 10.52 for the irrigation ditch, 15.85 for the riparian zone, 15.05 for the desert steppe, and 16.14 for the farmland. According to established threshold criteria, Koerselman and Arthur (1996) reported that N:P < 14 indicates N limitation, N:P > 16 indicates P limitation, and 14 < N:P < 16 signifies co-limitation by N and P. Güsewell (2004) proposed that N:P < 10 or > 20 characterizes short-term N or P limitation, respectively. These results demonstrate that plant growth conditions in the desert steppe are primarily co-limited by N and P (with N:P ratios in floodplain, desert steppe, and farmland all within the 14–16 range), thus aligning with Koerselman’s theoretical framework for nutrient limitation patterns in the study region.

The four habitats (irrigation ditches, riparian zones, desert steppe, and farmland) exhibited distinct abiotic and biotic characteristics that determined their dominant stress factors.

In the irrigation ditch habitats, the water supply is stable but subject to human disturbance (e.g., dredging) and nutrient enrichment (particularly phosphorus). *S. rostratum* thrives in this high-phosphorus environment by enhancing nitrogen use efficiency (high leaf C:N) to cope with nitrogen limitation while utilizing abundant phosphorus resources (low leaf C:P) to promote growth. Its stem acts as a “nutrient transit pool” that temporarily stores absorbed phosphorus and nitrogen for rapid transport to reproductive organs, thus ensuring efficient resource allocation for reproduction.

In the riparian zone habitats, hydrological conditions are unstable (flooding in wet seasons, drought in dry seasons), substrates are easily disturbed, nutrient cycling is rapid, and intermittent flooding-drought stress occurs. *S. rostratum* increases chlorophyll content to enhance photosynthesis, stores nutrients in roots, and prioritizes allocation to leaves and flowers, thus reflecting a reproduction-first strategy. Simultaneously, it alters soil nutrient cycling by increasing the C, N, and nitrate nitrogen content, reducing the C:N ratio, accelerating organic matter decomposition and nitrogen mineralization, and promoting ecosystem eutrophication to favor its own competition.

In the desert steppe habitats, the conditions are arid based on the low rainfall and high evaporation, the soil is nutrient poor, and area is affected by extreme drought and nitrogen and phosphorus co-limitation. *S. rostratum* exhibits high leaf C and P content, utilizing high carbon levels to synthesize structural or defensive compounds for stress resistance and high phosphorus to maintain metabolic efficiency under low-P conditions. The plant prioritizes allocating scarce resources to reproductive organs, thereby implementing a “reproduction-over-maintenance” strategy.

In the farmland habitats, intensive human management (e.g., plowing, fertilizing, irrigation, and harvesting) alters soil nutrients, creates pulsed resource availability, and causes frequent physical disturbance. *S. rostratum* directs large amounts of nutrients to reproductive organs, with its fruits showing significantly higher C, N, and P content and faster maturation compared to the other three habitats, thereby ensuring population continuity before the end of the agricultural cycle. Invasion by this plant significantly reduces soil nitrate nitrogen, likely through competitive uptake or alterations in microbial processes to consume readily available nitrogen sources, thereby inhibiting crop growth and reducing competition pressure.

As illustrated in **Figure 4**, this study systematically analyzed the dynamic responses of soil elements, including C, N, and P and their stoichiometric ratios (C:N, C:P, N:P) across diverse habitat types and invasion gradients of *S. rostratum*. The results demonstrated that under increasing invasion intensity, C, N, and P contents generally showed rising trends across habitats, with the riparian zones habitat exhibiting the most pronounced increases for all three elements. In contrast, the desert steppe habitat displayed slight decreases in these elemental contents. For the stoichiometric ratios, all habitats demonstrated an overall decreasing trend in C:N ratios, with the riparian zones habitat showing the most significant decline. Conversely, C:P and N:P ratios generally built up across habitats, again with the riparian zones habitat displaying the most substantial upward trends.

Research on the impact of *S. rostratum* invasion on soil characteristics reveals that as invasion intensity increases, significant changes occur in soil moisture content, C, N, and P contents, and their stoichiometric ratios in invaded areas. (1) Soil moisture dynamics: Soil water content generally decreases with increasing invasion intensity, aligning with findings by Cavaleri and Ma et al. (2021) and Te Beest et al. (2015), indicating higher water use efficiency in alien invasive species. This differential water utilization compared to native species alters soil water cycling processes (Vasquez-Valderrama et al., 2020). (2) C, N, and P elemental variations: Soil C, N, and P contents exhibit increasing trends with invasion intensity, demonstrating that plant invasions enhance soil nutrient accumulation (Liao et al., 2008), which is consistent with studies by Wang et al. (2021) and Vujanović et al. (2022) on invaded soil properties. While the soil ammonium nitrogen content does not show significant changes, the nitrate nitrogen content increases progressively with invasion intensity, leading to elevated levels of total nitrogen, ammonium nitrogen, and nitrate nitrogen in soils (Gornish and Thomas, 2015, Vujanović et al., 2022). Therefore, alien plant invasion has multifaceted and complex ecological effects on the soil nitrogen cycle. (3) Stoichiometric ratio changes: Soil C:N shows a downward trend as the invasion degree increases while C:P and N:P show an upward trend. This is consistent with findings on *Spartina alterniflora* invasion by Sha et al. (2021), who showed that this invasive plant increased the C:P and N:P values of shallow soil but did not significantly change the C:N ratio. Altered soil conditions promote the growth and development of alien invasive species while suppressing the growth of native species. This progressive alteration ultimately culminates in impaired ecosystem functioning, which is supported by the research of Sun et al. (2025).

The effects of *S. rostratum* invasion on soil properties showed significant habitat heterogeneity as follows. (1) Irrigation ditch: *S. rostratum* invasion significantly increased the soil C, N, and nitrate nitrogen contents and C:P and N:P, significantly reduced the water content, but did not significantly change the ammonium nitrogen, P, and C:N. (2) Riparian zones: *S. rostratum* invasion significantly increased the soil water, C, N, P, ammonium nitrogen, and nitrate nitrogen contents and C:P and N:P but significantly reduced the C:N. (3) Desert steppe habitat: *S. rostratum* invasion significantly increased soil water content, C, N, P, nitrate nitrogen, C:P, and N:P (P < 0.05). The C:N ratio exhibited a unimodal trend, with an initially decreasing followed by increasing trend with invasion intensity, while ammonium nitrogen content showed an inverse pattern (initial increase followed by decrease). (4) Farmland habitat: Invasion significantly elevated the water, C, N, and P contents and C:P and C:N (P < 0.05), significantly reduced the nitrate nitrogen, and did not change the ammonium nitrogen content and N:P. These findings validate the spatiotemporal heterogeneity theory of invasion ecological effects proposed by Ehrenfeld (2010) and align with the *S. alterniflora* invasion patterns reported by Sha et al. (2021) and Wang et al. (2021). This demonstrates that plant invasion impacts on soil properties are habitat-specific and likely attributable to distinct initial soil properties, hydrological regimes, and vegetation compositions across ecosystems.

The underlying reasons for this habitat heterogeneity may include three key points. Shift in dominant mechanisms: In impoverished habitats (such as desert steppe), the nitrogen efficiency strategy of *S. rostratum* itself is key to initiating invasion; whereas in nutrient-rich habitats (such as irrigation ditch and riparian zones), the “novel weapon” effect of its rhizosphere microorganisms plays a more central role in altering soil nutrient cycling. Modulation by moisture conditions: Water availability is a fundamental limiting factor for microbial activity and nutrient mineralization. Arid habitats constrain the role of microorganisms, thereby enhancing the innate strategies of the plant, whereas moist habitats provide a venue for microorganisms to function effectively. Boundary effect of initial soil conditions: The “starting point” at the beginning of the invasion determines the direction and intensity of the feedback. For instance, invasion leads to an increased N:P ratio in soils that were initially phosphorus-deficient, whereas the N:P ratio remains unchanged in phosphorus-rich farmland soils.

Analysis of the correlations between the contents of C, N, and P and their stoichiometric ratios in various organs of *S. rostratum* showed that they were jointly regulated by three factors: organ, invasion degree, and habitat type. At the organ level, C, N, and P and their stoichiometric ratios showed strong inter-organ synergy, indicating that *S. rostratum* may adapt to resource heterogeneity by adjusting the element allocation among organs under different environmental conditions.

Soil elemental analysis revealed robustly significant positive correlations among C, N, and P (P < 0.001), indicating synergistic mechanisms between soil organic matter accumulation and nutrient cycling. Further findings demonstrated that pH exerted regulatory effects. Soil pH showed robustly significant negative correlations with P, N, C, C:P and N:P but a positive correlation with C:N. This pattern aligns with Hinsinger (2001), confirming the classical theory of reduced phosphorus availability and altered organic matter decomposition rates in acidic environments. Moisture-driven dynamics were also observed. Soil water content correlated negatively with P and C:N but correlated positively with ammonium nitrogen. This suggests that moisture conditions likely modulate nitrogen-phosphorus transformation and bioavailability through indirect pathways, which was also confirmed by the study conducted by Muhammad et al. (2022). Environmental factor dominance revealed that both invasion intensity and habitat type exerted robustly significant effects (P < 0.001) on soil C, N, and P contents and their stoichiometric ratios, with habitat factors exhibiting significantly greater explanatory weight than invasion gradients.

This study employed PCA to reveal the divergence patterns of stoichiometric relationships in the *S. rostratum*-soil system across different habitats. The cumulative variance explained by the first two principal components exceeded 70% in all four habitats, indicating that the PCA effectively captured the core characteristics of variation and that the dominant underlying drivers differ fundamentally among habitats (**Figure 6**).

In the irrigation ditch habitat, positive correlations were observed between the soil C and N content, soil C:P and N:P ratios, plant P content, and plant C:N ratio, while negative correlations were found between the soil C:N and both plant P content and plant C:N ratio. This indicates a synergistic effect between soil organic matter accumulation and plant phosphorus uptake in this habitat. Soil organic matter likely enhances phosphorus availability by promoting microbial activity and phosphorus activation, while a higher soil C:N ratio may reflect relative nitrogen limitation, indirectly inhibiting plant phosphorus absorption and utilization. *S. rostratum* exhibited a “nutrient co-absorption strategy,” in which interactions with soil microorganisms were coordinated to optimize the homeostasis of carbon, nitrogen, and phosphorus stoichiometry, thereby supporting its rapid growth.

In the riparian zones habitat, negative correlations were observed between soil C:N, plant N and P content, and plant stoichiometric ratios (C:N, C:P, and N:P), as well as various soil nutrient indicators (C, N, P, C:N, N:P). This response pattern suggests that the riparian habitat is influenced by hydrological disturbances that lead to unstable resource availability. *S. rostratum* likely adopts a “resource allocation-prioritizing strategy” that preferentially allocates absorbed N and P elements to photosynthetic and reproductive organs such as flowers and leaves to cope with the dynamic environment and enhance fitness. Grime (1977) proposed that plants have evolved three primary strategies to cope with environmental pressures: competition (C) in fertile and low-disturbance environments, stress tolerance (S) under persistent stress, and rapid reproduction (R) in frequently disturbed habitats. Among these, the R-strategy supports the findings of this study.

In the desert steppe habitat, a negative correlation was found between the soil P and plant P content, high carbon contents were observed in the stems and leaves, relatively low P contents were observed in the fruit, but a high C:P ratio was observed. This indicates that in the arid desert grassland environment, *S. rostratum* adopts a strategy of optimizing carbon allocation and phosphorus conservation to enhance drought-resistant structures and sustain its growth. Simultaneously, the invasion of *S. rostratum* alters the nutrient cycling of the desert grassland, which is consistent with the process of its modification of the desert ecosystem’s nutrient cycling and corroborates the findings of Carey et al. (2015).

In the farmland habitat, negative correlations were observed between soil C:N and multiple soil indicators such as N and P, thus reflecting significant anthropogenic disturbances (e.g., fertilization and tillage) that profoundly affect the soil nutrient structure. In this habitat, the lowest P content (2.10 g/kg) was found in the leaves of *S. rostratum*, while a higher P content (3.33 g/kg) was recorded in the fruit. This suggests a tendency toward a “reproductive investment-prioritizing strategy” in which the proportion of phosphorus allocated to reproductive organs is increased to maximize reproductive output. Thus, *S. rostratum* exhibits stoichiometric plasticity and organ functional synergistic differentiation in response to highly disturbed and nutrient-rich environments.

In irrigation ditch habitats, where soil phosphorus content is relatively high and *S. rostratum* accumulates significant phosphorus in its stems, reducing sediment organic matter accumulation through regular desilting, planting deep-rooted species to compete for deep phosphorus resources, and blocking external phosphorus inputs (e.g., agricultural drainage) to control its invasion is recommended. In riparian zones habitats, frequent hydrological disturbances promote seed dispersal. Thus, measures such as establishing buffer zones to mitigate water level fluctuations, planting flood-tolerant native species (e.g., reeds and cattails) to form ecological barriers, and manually removing plants before and after flood seasons should be implemented. In desert steppe habitats, grazing intensity should be controlled to maintain vegetation cover, drought-tolerant native competitive species should be introduced, and degraded grasslands should be restored to enhance ecosystem resilience. In farmland habitats, human activities such as fertilization and irrigation promote the growth of *S. rostratum*. Thus, effective management strategies such as optimizing crop rotation systems, regularly removing weeds mechanically or manually, and strictly cleaning agricultural machinery should be implemented to prevent seed dispersal.

This study reveals that the soil N:P ratio increases with the degree of invasion by *S. rostratum*, particularly in riparian zones and farmland habitats. According to the threshold theory proposed by Koerselman and Arthur (1996), the leaf N:P ratios in different habitats in this study are 10.52 in irrigation ditches, 15.85 in riparian zones, 15.05 in desert grasslands, and 16.14 in farmland. This indicates that irrigation ditches may be nitrogen-limited, suggesting that native species might experience growth constraints due to nitrogen deficiency. In contrast, riparian zones, desert grasslands, and farmland are under nitrogen-phosphorus co-limitation or phosphorus limitation. *S. rostratum* gains a competitive advantage by adjusting its elemental allocation strategy, such as preferentially allocating phosphorus to flowers and fruits. This may lead to three long-term ecological effects. First is species screening and community structure changes. Such changes may occur because of long-term increases in the N:P ratio, which gradually eliminates species with high phosphorus demands. *S. rostratum*, with its high phosphorus use efficiency and reproductive investment, may gradually replace native species, leading to simplified community structures. Second is the reduced competitiveness of native species. Native species, particularly in phosphorus-limited environments, may experience further suppression in growth and reproduction, weakening their competitiveness. Third is ecosystem function degradation. Simplified community structures may impair ecosystem functions, such as nutrient cycling, water conservation, and biodiversity maintenance, especially in fragile habitats like desert steppe.

Studies have shown that plant-derived allelochemicals directly participate in and significantly regulate the rhizosphere pH environment. The secretion of many allelochemicals introduces protons into the soil, leading to changes in rhizosphere pH (Shi et al., 2025). This environment provides feedback that influences the dissociation state, adsorption behavior, and bioavailability of the allelochemicals themselves, and more critically, it reshapes the microbial habitat by selectively inhibiting or promoting the activity of specific functional microbial communities, thereby indirectly modulating various nutrient transformation processes driven by microorganisms. The litter and rhizosphere exudates of *S. rostratum* can impact surrounding plants and the soil environment.

Research by Scavo et al. (2019) confirmed that plant-derived allelochemicals directly participate in and significantly regulate the rhizosphere pH environment. The secretion of many allelochemicals releases protons into the soil, leading to a decrease in rhizosphere pH. This acidified environment provides feedback to influence the dissociation state, adsorption behavior, and bioavailability of the allelochemicals themselves, and more importantly, it selectively inhibits or promotes the activity of specific functional microbial communities, thereby reshaping the microbial habitat and indirectly regulating various microbially driven nutrient transformation processes. Studies by Shi et al. (2022) further demonstrated that secondary metabolites produced by the fungal community in the rhizosphere soil of *S. rostratum* exert allelopathic effects on both the plant itself and associated species. This study reveals that the content of soil organic matter varies across different habitats, resulting in differential effects of organic matter on allelochemical activity. Specifically, in habitats with higher organic matter content, the strong adsorption capacity of organic matter significantly reduces the activity of allelochemicals, thereby indirectly alleviating their inhibitory effects on nutrient availability and microbial communities. In contrast, in habitats with lower organic matter content, limited adsorption capacity allows allelochemicals to remain highly active, thereby exerting more pronounced influences on nutrient cycling and interspecific interactions. These findings highlight the critical role of soil organic matter in modulating allelopathic effects and provide new perspectives for understanding the invasion mechanisms of *S. rostratum* in diverse habitats.

Notably, this study has significant methodological limitations. All samples were collected at a single time point (August) and thus failed to capture dynamic changes throughout the growing season of *S. rostratum*. This limits the interpretability of physiological mechanisms and the reliability of long-term ecological inferences. Therefore, future research should incorporate multi-season sampling (summer and autumn) based on the growth status of *S. rostratum*, combine functional analyses of soil microbial communities, conduct controlled experiments to validate elemental allocation and competitive relationships, and employ techniques such as stable isotope labeling to trace elemental flows. These approaches will deepen the understanding of the invasion mechanisms of *S. rostratum* and optimize management strategies.The efficient elemental allocation mechanism of *S. rostratum* enables it to gain an advantage when competing with native species for limited nutrient resources, especially phosphorus. However, no indoor controlled experimental analysis has been conducted. Future research should incorporate controlled competition experiments to directly link the stoichiometric traits identified in this study with the competitive abilities of *S. rostratum* for light, water, and nutrients, thereby quantitatively elucidating its advantage in resource competition.

## Conclusion

This study elucidates the multiple intrinsic mechanisms behind the successful invasion of the alien plant *S. rostratum* by analyzing its ecological stoichiometric characteristics across different habitats.

Support for the Reproduction Priority Hypothesis and Organ-Specific Allocation HypothesisThe study found significant differences in the C, N, and P contents among different organs of S. rostratum. The reproductive organs (flowers and fruits) exhibited the highest N and P contents, while C was highly concentrated in the fruits. This indicates that S. rostratum adopts a “reproduction priority” strategy in resource allocation by preferentially directing limited and critical nutrient resources (such as N and P) to the reproductive process to ensure high seed yield and population expansion. Simultaneously, the distinct elemental allocation patterns observed in the roots, stems, leaves, flowers, and fruits provide evidence for the organ-specific allocation hypothesis, in which plants do not distribute resources uniformly but optimize allocation according to the specific functions of each organ.

Confirmation of the Ecological Stoichiometry Homeostasis HypothesisDespite significant habitat heterogeneity, the elemental ratios (e.g., C:N, C:P, and N:P) in the various organs of S. rostratum were maintained within a relatively stable range. This demonstrates that S. rostratum possesses strong ecological stoichiometric homeostasis, enabling it to resist external environmental fluctuations in nutrient availability through physiological regulation and maintain relatively stable internal elemental proportions, thereby ensuring normal growth and metabolic function. This homeostasis is key to its competitiveness in variable environments.

Support for the Habitat-Specific Response HypothesisThe stoichiometric characteristics of S. rostratum and its impact on the soil exhibited significant differences across the four habitats (irrigation ditches, riparian zones, desert steppe, and farmland). This supports the habitat-specific response hypothesis, indicating that the plant’s strategies and effects on the ecosystem are context-dependent and vary with local environmental conditions.

Support for the Plant-Soil Feedback HypothesisThe invasion of S. rostratum is not only influenced by the soil environment but also actively alters it. As the invasion intensifies, significant changes occur in soil physical and chemical properties, and these changes are habitat-specific. These modifications generally tend to create a soil environment more favorable for the growth of S. rostratum itself while being less conducive to the competition of native plants, thereby forming a positive feedback loop that drives the invasion process.

In conclusion, the successful invasion of *S. rostratum* is not determined by a single mechanism but rather by the synergistic effects of the mechanisms described by the multiple hypotheses above. Moreover, its inherent reproduction priority and organ-specific allocation strategies ensure efficient reproduction, its strong ecological stoichiometric homeostasis helps it resist environmental fluctuations, and its habitat-specific responses and strategic trade-offs enable niche optimization. In addition, it triggers positive plant-soil feedback to suppress competitors and alter ecosystem functions.

Although this study takes the Xinjiang landscape as a specific case, the ecological stoichiometry strategies of *S. rostratum* invasion revealed herein likely represent a universal adaptation model in arid and semi-arid environments. The conceptual framework and methodological system established in this study can serve as a reference for researching invasion mechanisms and management practices in other similar ecological regions. Future cross-regional comparative studies will help verify the universality of these strategies. Our findings may have broader implications for other semi-arid ecosystems. By uncovering the habitat-specific stoichiometric strategies employed by alien invasive species, these research results contribute to the field of global invasion ecology.

Future studies should expand the spatiotemporal scale (e.g., multi-season sampling and regional-scale validation), deepen the analysis of ecological stoichiometric mechanisms underlying *S. rostratum* invasion, and critically integrate physiological indicators, soil microbial functions, and competition experiments with native plants. Concurrently, quantifying the effects of anthropogenic disturbances (e.g., fertilization and irrigation) on resource allocation, developing habitat-sensitive invasion risk models, and advancing microbial-plant synergistic remediation technologies will collectively build an invasion management framework spanning from mechanisms to applications.

## Data Availability

The original contributions presented in the study are included in the article/supplementary material. Further inquiries can be directed to the corresponding author.
